# Gravitational waves in modified teleparallel theories of gravity

**DOI:** 10.1140/epjc/s10052-018-5967-x

**Published:** 2018-06-09

**Authors:** Habib Abedi, Salvatore Capozziello

**Affiliations:** 1 Department of Physics, University of Tehran, North Kargar Ave, Tehran, Iran; 2 Dipartimento di Fisica, Università di Napoli “Federico II”, Via Cinthia, 80126 Naples, Italy; 3 Istituto Nazionale di Fisica Nucleare (INFN), Sez. di Napoli, Via Cinthia, Naples, Italy; 4 Gran Sasso Science Institute, Via F. Crispi 7, 67100 L’Aquila, Italy; 5 Tomsk State Pedagogical University, Ul. Kievskaya, 60, Tomsk, 634061 Russia

## Abstract

Teleparallel theory of gravity and its modifications have been studied extensively in literature. However, gravitational waves has not been studied enough in the framework of teleparallelism. In the present study, we discuss gravitational waves in general theories of teleparallel gravity containing the torsion scalar *T*, the boundary term *B* and a scalar field $\phi$. The goal is to classify possible new polarizations generalizing results presented in Bamba et al. (Phys Lett B 727:194–198, arXiv:1309.2698, 2013). We show that, if the boundary term is minimally coupled to the torsion scalar and the scalar field, gravitational waves have the same polarization modes of General Relativity.

## Introduction

The observed late expansion of the universe can be described by either introducing an exotic form of energy (dark energy) or modifying gravity. In this framework, several modifications have been proposed [1–4] and, among them, the possibility to consider teleparallel gravity [5]. Einstein introduced the idea of teleparallelism soon after General Relativity (GR) [6]. Teleparallel Lagrangian coincides with Einstein-Hilbert Lagrangian up to a boundary term, *i.e.* $T=-R+B$, where *T* is the scalar torsion, *R* is the Ricci scalar and *B* is a boundary term. Therefore, GR and Teleparallel Equivalent General Relativity (TEGR) result in the same equations of motion.

However, difference between them arise in modified Lagrangians, where scalar fields coupled nonminimally to gravity or arbitrary functions of *T* or *R* are taken into account [5]. Such modifications of TEGR violate the local Lorentz symmetry invariance and result in six extra degrees of freedom [7]. In a more general case, the Lagrangian can be a function of both *T* and *R*, i.e. *f*(*T*, *R*) [8, 9]. This theory can be studied as *f*(*T*, *B*), where *B* is the boundary term [10, 11].

In both GR and TEGR, gravitational waves (GW) have two independent polarizations, usually denoted as plus and cross modes. However, extra polarizations appear in modified theories. The perturbation theory in the post-Minkowski limit is a way to study the number of GW polarizations.

The other way is the Newman-Penrose (NP) formalism [12, 13]. Adopting the NP formalism in a generic metric theory, plane GWs have six independent modes of polarization: considering the *z*-direction as the propagation direction of GWs, they are plus $(+)$, cross $(\times )$, breathing (*b*), longitudinal (*l*), vector-*x* (*x*) and vector-y (*y*) modes. These modes can be described by the independent NP quantities $\{ \Psi _2, \Psi _3, \Psi _4, \Phi _{22}\}$, where $\Psi _3$ and $\Psi _4$ are complex and each one describes two polarization modes. The extra polarization modes can be used to discriminate among modified theories of gravity beyond GR (see, e.g. Ref. [14, 15]). As shown in [16], GWs in *f*(*T*), and in its scalar-tensor representation, are equivalent to that in GR and TEGR [17]. In *f*(*R*) gravity, where the Lagrangian is an arbitrary function of Ricci scalar, three modes exist  [18–20]. Models $f(R, \Theta )$ and $f(R,\Theta ^\phi )$ were also studied in Ref. [21], where $\Theta$ and $\Theta ^\phi$ are the traces of the energy-momentum tensors of standard matter and of a scalar field, respectively. The Authors studied different form of function *f*, and have shown that the number of GW-modes depends on the form of it [22].

An important remark is necessary at this point. Modified theories of gravity are taken into account to achieve a comprehensive picture of cosmic dynamics ranging from early inflation, up to large scale structure formation and current acceleration of the universe [1–4]. The approach is aimed to give, in principle, a full geometric description of cosmic history consisting, for example, in extensions of GR, like *f*(*R*), or of TEGR, like *f*(*T*). The main task is explaining dynamics by further degrees of freedom of gravitational field (with respect to GR or TEGR) instead of invoking dark components [23]. However, to achieve a self-consistent description, further scalar fields could be necessary. For example, as discussed in [24], the flat rotation curve of galaxies is better fitted considering a theory like $f(R,\phi )$, instead of a pure *f*(*R*),because, in such a case, it is possible to reproduce the so-called Sanders potential with better precision. In this case, by a conformal transformation, it is shown that a model like $f(R,\phi )$ is analogue to $f(R,\Box R)$ so that the scalar field has a straightforward geometric interpretation too. In general, terms like $\Box R$, $\Box ^2R$ and so on appear as UV corrections that have effects also at IR scales (see [2] for a detailed discussion of this topic). In this perspective, further scalar fields, having a geometric or a matter origin, could be useful to describe coherently cosmic dynamics at any scale. Here, we consider TEGR extensions assuming not only general functions of the torsion scalar *T*, but also boundary terms *B* and a scalar field $\phi$ that, according to the discussion in [24], could be geometrically interpreted. In particular, considering further scalar fields is important for a full classification of GW modes and polarizations.

The present paper is organized as follows. The field equations of our modified teleparallel theory are derived in Sect. 2. Section 3 is devoted to study GWs in two modifications of teleparallel gravity; first, we study the case of scalar field nonminimally coupled to both the scalar torsion and the boundary term. Then, we assume a Lagrangian as a nonlinear function of the scalar torsion and the boundary term. In Sect. 4, we obtain the number of GW-polarizations when the scalar field kinetic term is coupled to the scalar torsion. We show that, due to the local Lorentz Invariance violation, such a coupling is not viable because of the extra degrees of freedom. In Sect. 5, we discuss the results and draw conclusions.

## Teleparallel gravity and its extensions

In teleparallel theories, vierbein fields describe gravity. Considering a set of orthonormal basis in each point of a generic manifold, the metric is given by

1 $$\begin{aligned} g_{\mu \nu }=\eta _{AB} e^A_{\mu } e^B_{\nu }, \end{aligned}$$

where $e^A_{\mu }$ are vierbein fields and $\eta _{AB}$ is the Minkowski metric. Then, one can write $e^A_{\mu } e_A^{\nu }=\delta ^\nu _\mu$. With the rule of absolute transport $\tilde{\nabla }_\mu e_A^{\nu }=0$, the Weitzenböck connection with vanishing Riemann tensor is defined by

2 $$\begin{aligned} \Gamma ^\alpha _{\nu \mu }:=e_A^{\alpha }\, \partial _\mu e^A_{\nu }. \end{aligned}$$

$\tilde{\nabla }_\mu$ is the covariant derivative is defined by the Weitzenbc̈k connection. This connection results in nonvanishing torsion tensor as follows

3 $$\begin{aligned} T^\alpha _{\mu \nu }=e_A^{\alpha }\left( \partial _\mu e^A_{\nu } - \partial _\nu e^A_{\mu } \right) . \end{aligned}$$

Defining contorsion and superpotential, respectively,

4 $$\begin{aligned} K^{\mu \nu }:= & {} -\frac{1}{2} \left( T^{\mu \nu }_{\rho }-T^{\nu \mu }_{\rho }-T_\rho ^{\mu \nu }\right) ,\end{aligned}$$

5 $$\begin{aligned} S_\rho ^{\mu \nu }:= & {} \frac{1}{2} \left( K^{\mu \nu }_{\rho }+\delta ^\mu _\rho T^{\alpha \nu }_{\alpha }-\delta ^\nu _\rho T^{\alpha \mu }_{\alpha }\right) , \end{aligned}$$

scalar torsion is

6 $$\begin{aligned} T:=S_\rho ^{\mu \nu } T^\rho _{\mu \nu }. \end{aligned}$$

The scalar torsion (6) is related to the Ricci scalar constructed by the Levi-Civita connection as follows

7 $$\begin{aligned} T=-R+B, \end{aligned}$$

where $B=2\nabla _\mu T_\nu ^{\nu \mu }$ is a boundary term in the teleparallel Lagrangian. If a scalar field is nonminimally coupled to the torsion scalar, the Einstein frame can be recovered by considering the boundary term *B* coupled to the scalar field [25]. Let us now take into account the following action

8 $$\begin{aligned} S= & {} \frac{1}{2}\int \mathrm{d}^4x \, e \big [ f(T,B, \phi )- \partial _\mu \phi \, \partial ^\mu \phi \nonumber \\&-2 V(\phi ) +2 \mathcal{L}_\mathrm{m} \big ] , \end{aligned}$$

where $e=\mathrm{det}\left( e^A_{\nu }\right) =\sqrt{-g}$, $V(\phi )$ is a generic potential and $\mathcal{L}_\mathrm{m}$ is the matter Lagrangian. The variation of action (8) with respect to the vierbein fields yields the following field equations

9 $$\begin{aligned}&e_A^{ \mu } \square f_B-e_A^{ \nu } \nabla ^\mu \nabla _\nu f_B\nonumber \\&\quad +\frac{1}{2} B f_B e_A^{ \mu } +2 \partial _\nu \left( f_B+f_T\right) S_A^{ \nu \mu } \nonumber \\&\quad +2e^{-1} \partial _\nu \left( e S_A^{ \nu \mu }\right) f_T -2f_T T^\alpha _{ \nu A} S_\alpha ^{\mu \nu } \nonumber \\&\quad -\frac{1}{2}e_A^{ \mu }\left[ f- \partial _\alpha \phi \, \partial ^\alpha \phi -2 V(\phi )\right] = \Theta _A^{\mu }, \end{aligned}$$

where $\Theta ^{\mu }_A=-\delta \mathcal{L}_\mathrm{m} / \delta h^A_{\mu }$ is the stress-energy tensor of matter. Equation (9) in spacetime indices become

10 $$\begin{aligned}&-f_T G_{\mu \nu } + \left( g_{\mu \nu } \square - \nabla _\mu \nabla _\nu \right) f_B\nonumber \\&\quad + \frac{1}{2} ( f_B B+ f_T T -f ) g_{\mu \nu } \nonumber \\&\quad + 2 S^{ \alpha }_{\nu \mu }\, \partial _\alpha (f_T+f_B) - g_{\mu \nu } \left[ \frac{1}{2} \partial _\alpha \phi \; \partial ^\alpha \phi + V(\phi ) \right] \nonumber \\&\quad + \partial _\mu \phi \; \partial _\nu \phi = \Theta _{\mu \nu }, \end{aligned}$$

where we have used

11 $$\begin{aligned} G^\nu _\sigma =-2 \left( e^{-1}\partial _\mu (eS^{\mu \nu }_A)-T^\rho _{\mu A}S^{ \nu \mu }_{\rho } - \frac{1}{4}e^{\nu }_A T\right) e^A_{ \sigma } . \end{aligned}$$

The variation of the action (8) with respect to the scalar field results in

12 $$\begin{aligned} \square \phi +\frac{1}{2} f^\prime -V^\prime =0 , \end{aligned}$$

where prime denotes the derivative with respect to the scalar field $\phi$. In the weak field approximation, the metric can be written as

13 $$\begin{aligned} g_{\mu \nu }=\eta _{\mu \nu }+h_{\mu \nu }, \end{aligned}$$

where $h_{\mu \nu }$ is small and first order, $\mathcal{O}\left( h^2\right) \ll 1$ with respect to the background. Thus, up to first order, one can write

14 $$\begin{aligned} e^A_{ \mu }=\delta ^A_{\mu }+h^A_{ \mu }. \end{aligned}$$

and

15 $$\begin{aligned} R^{(1)}_{\mu \nu }= & {} \frac{1}{2}\left( \partial _\rho \partial _\nu h^\rho _{\mu } +\partial ^\rho \partial _\mu h_{\nu \rho } - \square h_{\mu \nu } - \partial _\mu \partial _\nu h\right) ,\end{aligned}$$

16 $$\begin{aligned} R^{(1)}= & {} \partial _\rho \partial ^\mu h^\rho _{\mu } -\square h. \end{aligned}$$

where $h=\eta ^{\mu \nu }h_{\mu \nu }$ and $\square =\eta ^{\mu \nu } \partial _\mu \partial _\nu$. The indices are lowered and raised by the Minkowski background metric $\eta _{\mu \nu }$. The boundary term *B* is second order in perturbations; therefore, up to first order we have $R^{(1)}=-T^{(1)}$.

## Nonminimal coupling

### The role of scalar field

In order to develop our considerations, we can specify the function in (8) as

17 $$\begin{aligned} f(T,B,\phi ^i)=\left[ -1+\xi \, F(\phi )\right] T+ \chi \, E(\phi )\, B, \end{aligned}$$

where *F* and *E* are two arbitrary functions of scalar field. For $\xi =0=\chi$ it reduced to TEGR. Field equations get the following form

18 $$\begin{aligned} (- & {} 1+\xi F) G_{\mu \nu }+\chi \left( g_{\mu \nu }\square -\nabla _\mu \nabla _\nu \right) E \nonumber \\+ & {} 2S_{\nu \mu }^{\alpha } \partial _\alpha \left( \xi F+\chi E\right) -g_{\mu \nu } \left( \frac{1}{2} \partial _\alpha \phi \, \partial ^\alpha \phi +V\right) \nonumber \\+ & {} \partial _\mu \phi \, \partial _\nu \phi = \Theta _{\mu \nu }. \end{aligned}$$

At first order we have

19 $$\begin{aligned} (- & {} 1+\xi F_0) \left( R_{\mu \nu }^{(1)}-\frac{1}{2} \eta _{\mu \nu } R^{(1)}\right) \nonumber \\+ & {} \chi E_0^\prime \left( \eta _{\mu \nu } \partial ^2- \partial _\mu \partial _\nu \right) \delta \phi \nonumber \\- & {} h_{\mu \nu } V_0 - \eta _{\mu \nu } V^\prime _0 \, \delta \phi = \Theta _{\mu \nu }^{(1)}. \end{aligned}$$

Taking the trace of Eq. (19), we get

20 $$\begin{aligned} -(-1+\xi F_0)R^{(1)}+3\xi E^\prime _0 \square \delta \phi -hV_0 -4V^\prime _0 \, \delta \phi =\Theta ^{(1)} .\nonumber \\ \end{aligned}$$

According to these considerations, we can define

21 $$\begin{aligned}&\bar{h}_{\mu \nu }=h_{\mu \nu }-\frac{1}{2} \eta _{\mu \nu }h +\frac{\chi E^\prime _0}{-1+\xi F_0} \eta _{\mu \nu } \, \delta \phi ,\end{aligned}$$

22 $$\begin{aligned}&\bar{h}=-h +\frac{4\chi E^\prime _0}{-1+\xi F_0} \delta \phi ,\end{aligned}$$

23 $$\begin{aligned}&h_{\mu \nu }=\bar{h}_{\mu \nu }-\frac{1}{2} \eta _{\mu \nu }\bar{h} +\frac{\chi E^\prime _0}{-1+\xi F_0} \eta _{\mu \nu } \, \delta \phi , \end{aligned}$$

and, in vacuum, we have

24 $$\begin{aligned} \square \bar{h}_{\mu \nu }=0. \end{aligned}$$

With the plane wave ansätz, its solution in Fourier space is

25 $$\begin{aligned} \bar{h}_{\mu \nu }(\mathbf{k})= A_{\mu \nu }(\mathbf{k}) \, \exp \left( i k^\alpha x_\alpha \right) + \mathrm{c.c.} \end{aligned}$$

One can assume $\bar{\phi }$ as the minimum of the potential, *i.e.*

26 $$\begin{aligned} V\simeq V_0+\frac{1}{2} \gamma \, \left( \delta \phi \right) ^2 . \end{aligned}$$

The above scalar field equation, with the choice (17), gets the following form

27 $$\begin{aligned} \square \phi +\frac{1}{2} \left( \xi T F^\prime + \chi B E^\prime \right) -V^\prime =0 . \end{aligned}$$

At first order, it becomes

28 $$\begin{aligned} \square \delta \phi -\frac{1}{2} \xi F^\prime _0 R^{(1)}-V^{\prime \prime }_0 \delta \phi =0 , \end{aligned}$$

where we have used $B =\mathcal{O}(h^2)$ and $T^{(1)}=-R^{(1)}$. Then, we get

29 $$\begin{aligned} \left( \square -m^2\right) \delta \phi =0, \quad m^2=\frac{2V^{\prime \prime }_0 (-1+\xi F_0)}{2(-1+\xi F_0)-3\xi \chi F^\prime _0 E^\prime _0}, \end{aligned}$$

where $m^2$ defines an effective mass. We assumed $V^\prime _0=0$. The solution at first order is then

30 $$\begin{aligned} \delta \phi (\mathbf{q})= a(\mathbf{q}) \, \exp \left( iq^\alpha x_\alpha \right) + \mathrm{c.c.} \end{aligned}$$

Let us now consider *z* as the direction of wave traveling. Taking $\Omega$ as the angular frequency, we have

31 $$\begin{aligned} \mathbf{q}=\left( \Omega ,0,0,\sqrt{\Omega ^2-m^2}\right) , \end{aligned}$$

and the group velocity is

32 $$\begin{aligned} v_\mathrm{G} =\frac{\sqrt{\Omega ^2-m^2}}{\Omega } . \end{aligned}$$

Assuming the speed $v_G$ constant, we get

33 $$\begin{aligned} m=\sqrt{(1-v^2_\mathrm{G})}\Omega . \end{aligned}$$

The effect of gravitational polarization can be studied by the geodesic deviation,

34 $$\begin{aligned} \ddot{x}_i=-R_{itjt}x_j. \end{aligned}$$

Only the “electric part” of the Riemann tensor, i.e. $R_{itjt}$, affects the geodesic deviation. In absence of modes that are described by Eq. (25), i.e. $\bar{h}_{ij}=0$, we have

35 $$\begin{aligned} h_{\mu \nu }= \frac{\chi E_0^\prime }{-1+\xi F_0} \eta _{\mu \nu } \, \delta \phi . \end{aligned}$$

Then, geodesic deviation becomes

36 $$\begin{aligned} \ddot{x}_i=\frac{\chi E_0^\prime }{2\left( -1+\xi F_0\right) } \left( \eta _{ij} \, \ddot{\delta \phi }+ \left( \delta \phi \right) _{,ij}\right) x_j . \end{aligned}$$

Expressing (36) in components, one gets

37 $$\begin{aligned} \ddot{x}= & {} -\frac{\chi E_0^\prime \Omega ^2}{2\left( -1+\xi F_0\right) } \, \delta \phi \, x,\nonumber \\ \ddot{y}= & {} -\frac{\chi E_0^\prime \Omega ^2}{2\left( -1+\xi F_0\right) } \, \delta \phi \, y,\nonumber \\ \ddot{z}= & {} -\frac{\chi E_0^\prime m^2}{2\left( -1+\xi F_0\right) } \, \delta \phi \, z . \end{aligned}$$

If $\Omega \gg m$, the displacement in longitudinal direction is smaller than the transverse one, $\ddot{z}/z = \left( m/\Omega \right) ^2 \, \ddot{x}/x$. In very low frequency band, *l* and *b* modes can be of the same order. Considering the weak field limit, we can adopt the NP formalism. To obtain the independent NP quantities, one can use the solution (30), that is

38 $$\begin{aligned} R_{\mu \nu }^{(1)}= & {} \left[ \left( -\frac{1}{-1+\xi F_0}+\frac{3}{2} \right) \eta _{\mu \nu } \square \right. \nonumber \\&\left. + \frac{1}{-1+\xi F_0} \partial _\mu \partial _\nu \right] \chi E^\prime _0 \delta \phi . \end{aligned}$$

Defining a set of tetrads $\left( \mathbf{e}_t,\mathbf{e}_x, \mathbf{e}_y,\mathbf{e}_z \right)$, the null tetrads are

39 $$\begin{aligned} \begin{array}{lllllll} \mathbf{k}&{}=&{} \frac{1}{\sqrt{2}} (\mathbf{e}_t+\mathbf{e}_z),&{} \mathbf{l}&{}=&{} \frac{1}{\sqrt{2}} (\mathbf{e}_t-\mathbf{e}_z),\\ \mathbf{m}&{}=&{} \frac{1}{\sqrt{2}} (\mathbf{e}_x+ i\mathbf{e}_y),&{} {\bar{\mathbf{m}}}&{}=&{} \frac{1}{\sqrt{2}} (\mathbf{e}_x- i\mathbf{e}_y), \end{array} \end{aligned}$$

where $\mathbf{m}$ and ${\bar{\mathbf{m}}}$ are complex but $\mathbf{l}$ and $\mathbf{k}$ are real. The null tetrads satisfy following relations

40 $$\begin{aligned} -\mathbf{k}\cdot \mathbf{l}= & {} {\bar{\mathbf{m}}}\cdot \mathbf{m}=1,\nonumber \\ \mathbf{k} \cdot \mathbf{l}= & {} \mathbf{k}\cdot { \bar{\mathbf{m}}}= \mathbf{l} \cdot \mathbf{m} = \mathbf{l} \cdot {\bar{\mathbf{m}}} =0. \end{aligned}$$

Then the non-vanishing NP quantities become

41 $$\begin{aligned}&\Psi _4={-R_\mathbf{l}\bar{\mathbf{m}}{} \mathbf{l}\bar{\mathbf{m}}} \sim + \quad \text {and}\quad \times \quad \text {modes},\end{aligned}$$

42 $$\begin{aligned}&\Psi _3=-\frac{1}{2}{R_\mathbf{l} \bar{\mathbf{m}}} \sim x \quad \text {and}\quad y\quad \text {modes},\end{aligned}$$

43 $$\begin{aligned}&\Psi _2= \frac{1}{6} R_\mathbf{lk}\sim l \quad \text {mode},\end{aligned}$$

44 $$\begin{aligned}&\Phi _{22}= -\frac{1}{2} R_\mathbf{ll} \sim b \quad \text {mode}. \end{aligned}$$

Then, we have

45 $$\begin{aligned}&\Psi _3=0,\end{aligned}$$

46 $$\begin{aligned}&\Psi _2= \frac{\chi E^\prime _0 m^2 a \exp \left( iq_\alpha x^\alpha \right) }{12}\left[ \frac{1}{-1+\xi F_0}-\frac{3}{2} \right] \end{aligned}$$

47 $$\begin{aligned}&\Phi _{22}=-\frac{\chi E_0^\prime }{2(-1+\xi F_0)} \exp \left( iq_\alpha x^\alpha \right) \left( q_t-q_z\right) ^2 \end{aligned}$$

therefore, in general, we have four independent polarizations: $\times$, $+$, *b* and *l* modes. However, the NP formalism can be used for massless waves. Considering $V^{\prime \prime }_0=0$ we have

48 $$\begin{aligned} \Psi _2=&0=\Psi _3,&\Psi _4 \ne 0 \ne \Phi _{22} \end{aligned}$$

therefore there exists just three modes: $\times$, $+$ and *b*. The case in which $\chi =0$ results in $\Phi _{22}=0$, consequently, the two polarization modes of GR remain. Consider that these two polarizations are obtained also in TEGR. It is worth noticing that the massless scalar field, coupled with the boundary term, leads to the breathing mode.

### The *f*(*T*, *B*) theory

Let us consider now the following action

49 $$\begin{aligned} S=\frac{1}{2} \int \mathrm{d}^4x \, e \, f(T,B). \end{aligned}$$

The field equations are

50 $$\begin{aligned}&-f_T G_{\mu \nu }+\left( g_{\mu \nu } \square - \nabla _\mu \nabla _\nu \right) f_B\nonumber \\&\quad +\frac{1}{2} g_{\mu \nu } \left( f_B B+f_T T -f\right) \nonumber \\&\quad +2 S^{\alpha }_{\nu \mu } \, \partial _\alpha \left( f_T+f_B\right) =0. \end{aligned}$$

Supposing *f*(*T*, *B*) being an analytic function of *T* and *B*, one can expand it as follows

51 $$\begin{aligned} f(T,B)= & {} f(T_0,B_0)+f_T(T_0,B_0)\, T+ f_B(T_0,B_0) \, B \nonumber \\&+ f_{TB}(T_0,B_0)\, TB + \cdots . \end{aligned}$$

Then the field equations at first order become

52 $$\begin{aligned} -f_{T_0} G_{\mu \nu }^{(1)}+ f_{T_0 B_0} \left( \eta _{\mu \nu } \square - \partial _\mu \partial _\nu \right) T^{(1)}=0. \end{aligned}$$

Up to first order we have again $R^{(1)}=-T^{(1)}$. Therefore, we get

53 $$\begin{aligned} f_{T_0}\left( R^{(1)}_{\mu \nu }-\frac{1}{2}\eta _{\mu \nu } R^{(1)}\right) +f_{T_0 B_0} \left( \eta _{\mu \nu } \square - \partial _\mu \partial _\nu \right) R^{(1)}=0.\nonumber \\ \end{aligned}$$

Using the transformation

54 $$\begin{aligned} h_{\mu \nu }=\bar{h}_{\mu \nu }-\frac{1}{2}\bar{h} \eta _{\mu \nu } -\frac{f_{T_0 B_0}}{f_{T_0}} R^{(1)} \eta _{\mu \nu }, \end{aligned}$$

we get

55 $$\begin{aligned} \square \bar{h}_{\mu \nu }=0. \end{aligned}$$

The trace of Eq. (52) is

56 $$\begin{aligned} f_{T_0} R^{(1)} -3f_{T_0 B_0} \square R^{(1)}=0. \end{aligned}$$

Then we have

57 $$\begin{aligned} \square R^{(1)}+m^2 R^{(1)}=0 , \end{aligned}$$

where

58 $$\begin{aligned} m^2=-\frac{f_{T_0}}{3f_{T_0 B_0}}, \end{aligned}$$

is the effective mass. The solution of this equation is

59 $$\begin{aligned} R^{(1)}= \hat{R}\left( q^\rho \right) \, \exp \left( i q_\rho x^\rho \right) . \end{aligned}$$

One can study different cases:

- If $f_{T_0 B_0}=0$ (for example $F(T)+G(B)$), then, from Eq. (56), we get
  60 $$\begin{aligned} R^{(1)}=0. \end{aligned}$$
- In order to respect the local Lorentz symmetry invariance, we have to consider $f(T,B)=F(R)$. In this case, the field equations reduce to
  61 $$\begin{aligned} F_RG_{\mu \nu }+ & {} \left( g_{\mu \nu } \square - \nabla _\mu \nabla _\nu \right) F_R\nonumber \\+ & {} \frac{1}{2} g_{\mu \nu } \left( F_RR-F\right) =0. \end{aligned}$$
  By considering a situation similar to the paper [26],
  62 $$\begin{aligned} F(R)=R+\alpha R^2+ \beta R^3, \end{aligned}$$
  the mass (58) reduces to $m^2=-\frac{1}{6\alpha }$ and then results for *F*(*R*) gravity can be easily recovered.

Furthermore, the action (49) can be written as

63 $$\begin{aligned} S\,=\,\frac{1}{2}\int \mathrm{d}^4x \, e \left[ f_{,\phi }T+f_{,\psi }-2U(\phi ,\psi )\right] , \end{aligned}$$

where the new potential is $2U(\phi ,\psi )=f_{,\phi }+f_{,\psi }\psi -f(\phi ,\psi )$. Varying the action with respect to $\phi$ and $\psi$ by assuming $f_{,\phi \phi }\ne 0$ and $f_{,\psi \psi } \ne 0$, we get the identifications $\phi =T$ and $\psi =B$ that can be used as Lagrange multipliers, that is

64 $$\begin{aligned} \begin{array}{lll} S&{}=&{}\frac{1}{2}\int \mathrm{d}^4x \,e \, \left[ f+f_{,\phi }\left( T-\phi \right) +f_{,\psi }\left( B-\psi \right) \right] \nonumber \\ &{}=&{} \frac{1}{2}\int \mathrm{d}^4x \,e \, \left[ f-f_{,\phi } \left( {}^{(3)}R+\phi \right) -f_{,\psi }\psi \right. \nonumber \\ &{}&{}\left. - f_{,\phi } \left( \bar{\Sigma }^{ij} \bar{\Sigma }_{ij} - \bar{\Sigma }^2\right) + (f_{,\phi }+f_{,\psi })\mathcal{D}_T+ f_{,\psi } \mathcal{D}_R\right] .\nonumber \end{array}\\ \end{aligned}$$

Finally, we get

65 $$\begin{aligned} S= & {} \int \mathrm{d}^4x \, N \sqrt{h} \Bigg \{\frac{1}{2} f-\frac{1}{2} f_{,\phi } \left( {}^{(3)}R+\phi \right) \nonumber \\&-\frac{1}{2} f_{,\psi } \psi -\frac{1}{2} f_{,\phi } \left( \bar{\Sigma }^{ij} \bar{\Sigma }_{ij} -\bar{\Sigma }^2\right) \nonumber \\&+\frac{\bar{\Sigma }}{N} \left( N_j \bar{D}^j f_\psi - f_{,\psi \psi } \dot{\psi }-f_{,\psi \phi }\dot{\phi }\right) \nonumber \\&\times \bar{D}_jf_\psi \, \bar{D}^j \ln N+ h^{ij} T^\alpha _{j \alpha }\, \bar{D}_i(f_{,\phi }+f_{,\psi })\nonumber \\&- \bar{D}_j (f_{,\phi }+f_{,\psi }) \, \bar{D}^j \ln N+ A^\mu \, \nabla _\mu (f_{,\phi }+f_{,\psi }) \Bigg \}, \end{aligned}$$

where

66 $$\begin{aligned} A^\mu= & {} n^\mu \, \bar{D}_i \omega ^i+ \frac{n^\mu }{N} \bar{D}_i\left( N^b B^i_{b}\right) \nonumber \\&+ \frac{n^\mu }{2}\left( B^{ij} \, \bar{D}_j \omega _i+ \omega _j \, \bar{D}_i B^{ji}\right) . \end{aligned}$$

We have used the integration by parts. One can simply write the momentum conjugates of degrees of freedom as

67 $$\begin{aligned} \pi ^\phi= & {} \frac{\partial S}{\partial \dot{\phi }}=\sqrt{h} \left[ -\bar{\Sigma } f_{,\psi \phi } +A^0 N\left( f_{,\phi \phi }+f_{,\psi \phi }\right) \right] , \end{aligned}$$

68 $$\begin{aligned} \pi ^\psi= & {} \frac{\partial S}{\partial \dot{\psi }} =\sqrt{h} \left[ -\bar{\Sigma } f_{,\psi \psi } +A^0 N \left( f_{,\phi \psi }+f_{,\psi \psi }\right) \right] , \end{aligned}$$

69 $$\begin{aligned} \pi ^N=&\frac{\partial S}{\partial \dot{N}}=0, \quad \pi ^{N^i}=\frac{\partial S}{\partial \dot{N}^i}=0. \end{aligned}$$

The only term that contains time derivative of teleparallel extra degrees of freedom is the second one in the second line of the action; according to our definition of torsion, we have

70 $$\begin{aligned} T^\alpha _{j \alpha }= & {} T^0_{j 0}+T^i_{j i }=-\frac{1}{N} \partial _j\left( N+N^a \omega _a\right) \nonumber \\&+\frac{\omega _a}{N} \partial _j \left( N^a+\omega ^a N + N^b B^a_{b} \right) \nonumber \\&+\,\frac{1}{N} \partial _0\omega _j -\frac{\omega _a}{N} \partial _0 \left( h^a_{j}+B^a_{j}\right) \nonumber \\&+ \left( \omega ^i+\frac{N^i}{N}\right) \left( \partial _j \omega _i - \partial _i \omega _j\right) \nonumber \\&+ \left( B^i_a+\frac{N^i}{N} \omega _a +h^i_a\right) \nonumber \\&\times \,\left[ \partial _i\left( h^a_j+B^a_j\right) -\partial _j\left( h^a_i+B^a_i\right) \right] . \end{aligned}$$

Then, using

71 $$\begin{aligned} \frac{\partial T^\alpha _{j \alpha }}{\partial \dot{\omega }_k}=&\frac{1}{N} \delta ^k_j , \quad \frac{\partial T^\alpha _{j \alpha }}{\partial \dot{B}^b_k}= -\frac{\omega _b}{N} \delta ^k_j. \end{aligned}$$

we have

72 $$\begin{aligned} \pi ^{\omega _k}= & {} \frac{\partial S}{\partial \dot{\omega }_k}= \sqrt{h} h^{ik} \bar{D}_i\left( f_{,\phi }+f_{,\chi }\right) ,\end{aligned}$$

73 $$\begin{aligned} \pi ^{B^a_k}= & {} \frac{\partial S}{\partial \dot{B}^b_k}= -\sqrt{h} h^{ik}\omega _a \bar{D}_i\left( f_{,\phi }+f_{,\chi }\right) . \end{aligned}$$

The momentum conjugate of $h_{ij}$ becomes

74 $$\begin{aligned} \pi ^{kl}= & {} \frac{\partial S}{\partial \dot{h}_{kl}}=\frac{\sqrt{h}}{2}\bigg [ -f_{,\phi }\left( \bar{\Sigma }^{kl}-h^{kl} \bar{\Sigma } \right) \nonumber \\&+\frac{h^{kl}}{N} \left( N_j \bar{D}^j f_{,\psi } - f_{,\psi \psi } \dot{\psi }- f_{,\psi \phi } \dot{\phi }\right) \nonumber \\&-2 h^{ik} h^{al} \omega _a \bar{D}_i \left( f_{,\phi }+f_{,\psi }\right) \bigg ]. \end{aligned}$$

It is worth noticing that quantities constructed from $h_{ij}$ do not contain any extra degrees of freedom. Its trace becomes

75 $$\begin{aligned} \pi= & {} \frac{\sqrt{h}}{2} \bigg [2f_{,\phi }\bar{\Sigma }+\frac{3}{N} \left( N_j \bar{D}^j f_{,\psi }-f_{,\psi \psi } \dot{\psi } - f_{,\psi \phi } \dot{\phi } \right) \nonumber \\&- 2 h^{al}\omega _a \bar{D}_i \left( f_{,\phi }+f_{,\psi }\right) \bigg ]. \end{aligned}$$

In summary, we have classified all possible momenta related to the degrees of freedom.

## Kinetic coupling

In action (8) we have considered that gravity couples minimally to kinetic term. In this section, we study such coupling in view of GW polarizations. Let us consider the ADM line element,

76 $$\begin{aligned} \mathrm{d}s^2=-N^2 \, \mathrm{d}t^2+ h_{ij}\left( \mathrm{d}x^i+N^i \, \mathrm{d}t \right) \, \left( \mathrm{d}x^j+N^j \, \mathrm{d}t \right) , \end{aligned}$$

where *N*, $N^i$ and $h_{ij}$ are the lapse function, the shift function and the metric of three-dimensional space, respectively. One can write extrinsic curvature as follows

77 $$\begin{aligned} \bar{\Sigma }_{ij}=\frac{1}{N}\left( \dot{h}_{ij} -\bar{D}_i N_j-\bar{D}_jN_i \right) . \end{aligned}$$

where $\bar{D}_i$ the 3-Levi-Civita covariant derivative. Then the Ricci scalar is given by

78 $$\begin{aligned} R=&{}^{(3)}R+ \bar{\Sigma }^{ij} \bar{\Sigma }_{ij} - \bar{\Sigma }^2 +\mathcal{D}_R, \end{aligned}$$

where $\bar{\Sigma }=\bar{\Sigma }^{ij} h_{ij}$ is the trace of the extrinsic curvature and

79 $$\begin{aligned} \mathcal{D}_R&=\frac{2}{N \sqrt{\gamma } } \partial _t \left( \sqrt{\gamma }\bar{\Sigma } \right) - \frac{2}{N}\bar{D}_i \left( \bar{\Sigma } N^i+\gamma ^{ij} \partial _j N \right) . \end{aligned}$$

In GR, *R* coupled to $\partial _\mu \phi \, \partial ^\mu \phi$ changes the number of dynamical degrees of freedom (see [27] for a discussion). In view of this, let us onsider the action with the following term

80 $$\begin{aligned} S\supset \int \mathrm{d}^4x \, \sqrt{-g} R X, \end{aligned}$$

where $X=\frac{1}{2} \partial _\alpha \phi \, \partial ^\alpha \phi$ is the kinetic term. By using the ADM decomposition, we have

81 $$\begin{aligned} X=-\frac{1}{2N^2} \dot{\phi }^2+\frac{N^i}{N^2} \dot{\phi }\, \partial _i \phi + \frac{1}{2} \left( h^{ij}-\frac{N^i N^j}{N^2}\right) \partial _i \phi \, \partial _j\phi ,\nonumber \\ \end{aligned}$$

and then the action contains the following term

82 $$\begin{aligned} S&\supset \int \mathrm{d}^4x \, \sqrt{-g} \left( -\frac{1}{2N^2} \dot{\phi }^2 \right) \, \left[ -2\nabla _\mu (\bar{\Sigma }n^\mu ) \right] \nonumber \\&\quad = \int \mathrm{d}^4x \, \frac{\dot{\phi }^2 \dot{\bar{\Sigma }}}{N^2}. \end{aligned}$$

According to this development, the lapse function is a dynamical variable. Therefore, it is unstable and hence not viable for GWs. However, some fine tuned combination of geometry and scalar field derivatives exists which includes $G^{\mu \nu } \partial _\mu \phi \partial _\nu \phi$ where $G^{\mu \nu }$ is the Einstein tensor (see [28]). These extra degrees of freedom cancel out and allow the models to be stable and avoiding the Ostrogradskij instability. In the teleparallel approach, the vierbein fields, related to the ADM line element (76) can be written as [29]

83 $$\begin{aligned} \begin{array}{llllll} e^0_{\mu }&{}=&{} (N , \mathbf{0}),&{} e^a_{\mu }&{}=&{} (N^a , h^a_{i}),\\ e_0^{\mu }&{}=&{} (1/N , -N^i/N),&{} e_a^{\mu }&{}=&{} (0 , h_a^{i}). \end{array} \end{aligned}$$

The torsion becomes

84 $$\begin{aligned} T=&-{}^{(3)}R- \bar{\Sigma }^{ij} \bar{\Sigma }_{ij} + \bar{\Sigma }^2 +\mathcal{D}_T , \end{aligned}$$

where [29]

85 $$\begin{aligned} \mathcal{D}_T\,=-\frac{2}{N}\bar{D}_k(N T^{i k}_{ i}) . \end{aligned}$$

is the boundary terms in *T*. Therefore we can split *B* in a curvature and torsion component, that is

86 $$\begin{aligned} B=\mathcal{D}_R+\mathcal{D}_T. \end{aligned}$$

Clearly $\mathcal{D}_T$ has no time derivative while $\mathcal{D}_R$ contains time derivative of $\bar{\Sigma }$. This means, in general, that the boundary term *B* contains time derivative. One can conclude that the coupling of $\mathcal{D}_R$ or *B* to the kinetic term will result in instability.

Let us consider now the following action

87 $$\begin{aligned} S= & {} \int \mathrm{d}^4x\, e \left[ R+\frac{1}{2}\partial _\mu \phi \, \partial ^\mu \phi -V(\phi ) \right. \nonumber \\&\left. + \frac{1}{2}(\xi T + \chi B) \partial _\mu \phi \, \partial ^\mu \phi \right] , \end{aligned}$$

where $\xi$ and $\chi$ represent coupling constant to the torsion scalar and the boundary term. $\xi +\chi =0$ is the case that has been studied in Ref. [28], then it was assumed $\chi =\xi =0$. However, in action (87), it is enough to consider $\chi =0$, in order to avoid ghost instabilities. The action we are going to study contains a torsion scalar nonminimaly coupled to the kinetic term as follows

88 $$\begin{aligned} S= & {} \int \mathrm{d}^4x\, e \left[ -\frac{T}{2}(1+\xi \partial _\mu \phi \, \partial ^\mu \phi )+\frac{1}{2}\partial _\mu \phi \, \partial ^\mu \phi \right. \nonumber \\&-V(\phi )+\mathcal{L}_\mathrm{m} \bigg ]. \end{aligned}$$

For $\xi =0$, the action (88) is equivalent to GR minimally coupled to a scalar field. Varying with respect to the vierbein fields yields

89 $$\begin{aligned}&-2\left( 1+\xi \partial _\mu \phi \, \partial ^\mu \phi \right) \left[ e^{-1}\partial _\alpha \big (e S^{ \alpha \nu }_{A} \big )-T^{\rho }_{ \beta A} S^{ \nu \beta }_\rho \right] \nonumber \\&\quad +\frac{1}{2} e^{\nu }_A T- 2 \xi S^{ \alpha \nu }_A \partial _\alpha \big (\partial _\gamma \phi \, \partial ^\gamma \phi \big ) \nonumber \\&\quad -e^{\nu }_A \left[ \frac{1}{2} \partial _\gamma \phi \, \partial ^\gamma \phi - V(\phi )\right] +\partial ^\nu \phi \, \partial _A \phi =\Theta ^{\nu }_A . \end{aligned}$$

contracting with $e^A_{\sigma }$, we get

90 $$\begin{aligned}&G^\nu _\sigma \left( 1+\xi \partial _\mu \phi \, \partial ^\mu \phi \right) - 2 \xi S^{ \alpha \nu }_\sigma \partial _\alpha \big (\partial _\gamma \phi \, \partial ^\gamma \phi \big ) \nonumber \\&\quad -\delta ^\nu _{\sigma } \left[ \xi T \partial _\gamma \phi \, \partial ^\gamma \phi +\frac{1}{2} \partial _\gamma \phi \, \partial ^\gamma \phi - V(\phi ) \right] \nonumber \\&\quad +\partial ^\nu \phi \, \partial _\sigma \phi =\Theta ^\nu _{\sigma } . \end{aligned}$$

The trace of Eq. (89) is

91 $$\begin{aligned}&-2 (1+ \xi \partial _\mu \phi \, \partial ^\mu \phi ) \left[ e^{-1} e^A_{\, \mu } \partial _\alpha (eS^{\, \alpha \nu }_A) +T \right] +2 T \nonumber \\&\quad -2 \xi S^{\, \alpha \nu }_\nu \, \partial _\alpha (\partial _\gamma \phi \, \partial ^\gamma \phi ) -\partial _\gamma \phi \, \partial ^\gamma \phi +4 V= \Theta . \end{aligned}$$

This modification is not local Lorentz invariant. Variation of the action with respect to the scalar field also results in

92 $$\begin{aligned} \square \phi +V_{,\phi }=\xi \, \partial ^\mu \phi \, \partial _\mu T . \end{aligned}$$

Action (88) has been studied in Ref. [30]. In a Friedman-Robertson-Walker background, we have $T=G_{00}=6H^2$. This implies that the derivative coupling $T \partial _\mu \phi \, \partial ^\mu \phi$, on such a background, gives the same cosmological evolution as the derivative coupling of the scalar field to the Einstein tensor $G^{\mu \nu } \partial _\nu \phi \, \partial _\mu \phi$. However, beyond background level, they will differ (see also [31]). Equations (90) and (92), at first order, results in

93 $$\begin{aligned} \left[ G_\sigma ^{\nu } -\delta ^\nu _\sigma \left( \frac{1}{2} \partial _\gamma \phi \, \partial ^\gamma \phi -V(\phi )\right) + \partial ^\nu \phi \, \partial _\sigma \phi \right] ^{(1)} = \left( \Theta ^\nu _\sigma \right) ^{(1)}, \end{aligned}$$

and

94 $$\begin{aligned} \left( \square \phi +V_{,\phi }\right) ^{(1)}=0. \end{aligned}$$

These equations are exactly the same as equations of motion for a scalar field minimally coupled to the Ricci scalar. Therefore, the number of GW polarizations are the same as in the Einstein gravity.

Under local Lorentz transformation $e^A_{\mu }=\Lambda ^A_{B}\left( x^\nu \right) \, e^B$, some quantities of teleparallel gravity are not invariant, *e.g.* torsion tensor becomes $T^\alpha _{\mu \nu }+\Lambda _B^{A} e_A^{\alpha } \left( e^C_{\nu } \partial _\mu - e^C_{\mu } \partial _\nu \right) \Lambda _C$. The infinitesimal local Lorentz transformation is $\Lambda ^A_{\phantom {A}B}(\mathbf{x})=\left( e^\omega \right) ^A_{\phantom {A}B} \simeq \delta ^A_{\phantom {A}B}+\omega ^A_{\phantom {A}B}$. By breaking this symmetry, six extra degrees of freedom appear [32], i.e.

95 $$\begin{aligned} \omega ^0_{B}=&(0,\omega _B),\quad \omega ^a_{B}=&(\omega ^a, B^a_{b}), \end{aligned}$$

where $B^a_{b}$ is antisymmetric. Considering these new degrees of freedom, the vierbein fields (83), up to first order, get the following form

96 $$\begin{aligned} e^0_{\mu }= & {} (N+N^a \omega _a , \, \omega _i),\nonumber \\ e^a_{\mu }= & {} (N^a+N \omega ^a+N^b B^a_{b} ,\,h^a_{i}+B^a_{i}), \nonumber \\ e_0^{\mu }= & {} (1/N , \, -N^i/N-\omega ^i), \nonumber \\ e_a^{\mu }= & {} \left( -\omega _a/N ,\, h_a^{i}+B^i_{a}+\frac{N^i}{N}\omega _a\right) . \end{aligned}$$

Up to second order in extra degrees of freedom, after some simple calculations, one gets [32]

97 $$\begin{aligned} T=- & {} {}^{(3)}R+ \bar{\Sigma }^2-\bar{\Sigma }^{ij} \bar{\Sigma }_{ij}+ \frac{2}{N} \bar{D}_i \bar{D}^i N \nonumber \\- & {} \frac{2}{N} \bar{D}_i \left( h^{ij} N T^\alpha _{j\alpha }\right) \nonumber \\- & {} 2 \bar{\nabla }_\mu \left[ n^\mu \, \bar{D}_i \omega ^i + \frac{n^\mu }{N} \bar{D}_i (N^bB^i_{b})\right] \nonumber \\- & {} \bar{\nabla }_\mu \left[ n^\mu (B^{ij}\bar{D}_j \omega _i+\omega _j \bar{D}_i B^{ij})\right] . \end{aligned}$$

Now, let us consider an action with the following coupling term

98 $$\begin{aligned} \int \mathrm{d}^4x \, e T X. \end{aligned}$$

The action contains

99 $$\begin{aligned} S&\supset&\int \mathrm{d}^4x \frac{\dot{\phi }^2}{N^2} \, \partial _0\bigg [\frac{\bar{D}_i \omega ^i}{N}+\frac{\bar{D}(N^b B^i_{b})}{N^2}+\frac{B^{ij}\bar{D}_j\omega _i}{N} \nonumber \\&+\frac{\omega _j \, \bar{D}_iB^{ij}}{N}\bigg ]. \end{aligned}$$

Therefore, on considering extra degrees of freedom, the torsion scalar coupled into kinetic term results in instability.

### $G_{\mu \nu }$ coupled to field derivatives

Finally, let us consider the action containing the following term

100 $$\begin{aligned} G_{\mu \nu } \, \nabla ^\mu \phi \, \nabla ^\nu \phi . \end{aligned}$$

At first order, we get

101 $$\begin{aligned}&\left( \square -m^2\right) \delta \phi =0,\end{aligned}$$

102 $$\begin{aligned}&R^{(1)}_{\mu \nu }-\frac{1}{2} \eta _{\mu \nu } R^{(1)} =0. \end{aligned}$$

where the massive term is $m^2=V_{,\phi _0 \phi _0}$. Therefore, the number of GW polarization is the same as in GR plus a scalar mode related to the presence of the scalar field.

## Conclusions

The number of GW polarizations depends on the considered theory of gravity. In present work we have studied GWs in extended teleparallel gravity where a boundary term *B* and a further scalar field $\phi$ are taken into account beside the torsion scalar *T*. The conclusions we reached are the following. There is no extra polarization in TEGR and in *f*(*T*) theory with respect to GR as already shown in [16]. Here we demonstrated that a scalar field, non-minimally coupled to torsion, has only the two polarization of GR plus the scalar mode related to the scalar field itself. However, new polarizations appear when the scalar field is coupled to the boundary term *B*, beside the standard two modes of GR. One can also write the Lagrangian as a function of scalar torsion *T* and Ricci scalar *R*, however in order to study GW polarizations, it is better to decouple the Ricci scalar $R=-T+B$ and then using *f*(*T*, *B*). In *f*(*T*, *B*), extra massless and massive modes arise when the scalar torsion and the boundary term are non-minimally coupled as in the theory of $f(R)=f(-T+B)$. The detection of these extra modes could be a fundamental feature to discriminate between metric and teleparallel approaches (see [5] for a discussion).

In this perspective, the GW170817 event [33] has set important constraints and upper bounds on viable theories of gravity. In fact, besides the multi-messenger issues, the event provides constraints on the difference between the speed of electromagnetic and gravitational waves. This fact gives a formidable way to fix the mass of further gravitational modes which results very light (see [34] for details). Furthermore the GW170817 event allows the investigation of equivalence principle (through Shapiro delay measurement) and Lorentz invariance. The limits of possible violations of Lorentz invariance are reduced by the new observations, by up to ten orders of magnitude [34]. This fact is extremely relevant to discriminate between metric and teleparallel formulation of gravitational theories. Finally, GW170817 seems to exclude some alternatives to GR, including some scalar-tensor theories like Brans–Dicke gravity, Horava–Lifshitz gravity, and bimetric gravity [35]. Considering the present study, the reported data seem in favor of the tensor modes excluding the scalar ones. This means that *f*(*T*) gravity, showing the same gravitational modes as GR [16], should be favored with respect to other teleparallel theories involving further degrees of freedom. Starting from these preliminary results, it seems possible a complete classification of modified theories by gravitational waves. However, more events like GW170817 are necessary in order to fix precisely gravitational parameters and not giving just upper bounds. In this context, the present study could constitute a sort of paradigm in order to classify gravitational modes and polarizations (see also [14, 15]). In a forthcoming paper, the comparison with gravitational wave data will be developed in detail.
